# Capecitabine-induced radiation recall phenomenon: a case report

**DOI:** 10.12688/f1000research.1-64.v2

**Published:** 2013-03-07

**Authors:** José Aguilar, Elena García, Elisa García-Garre

**Affiliations:** 1 Breast Unit, Morales Meseguer Hospital, SMS (Murcia’s Health Service), Murcia, 30008, Spain

## Abstract

Radiation recall dermatitis is defined as an inflammatory reaction of the skin at the site of previous irradiation. Different drugs have been associated with triggering this phenomenon, and it can also affect other areas and organs where previous radiotherapy has been administered. The time gap between the inflammatory reaction and previous radiation can range from days to several years. We report a case of capecitabine-induced Radiation Therapy Oncology Group (RTOG) grade 4 (ulcerating dermatitis) recall skin toxicity of skin irradiated 3 years previously. To our knowledge, this is the first reported case of capecitabine-induced RTOG grade 4 (ulcerating dermatitis) recall skin toxicity of previously irradiated skin. Clinicians should be aware of this phenomenon, even when considering patients for whom it has been a long time since previous radiation therapy. This unusual and late drug side effect should be borne in mind in the differential diagnosis and management of advanced-disease patients as it may be confused with local relapse or infectious complication of previously operated areas.

## Introduction

We report a case of radiation recall phenomenon after the administration of capecitabine, consisting of pain, hyperpigmentation, and ulceration in the field of post-mastectomy irradiation (which the patient received 3 years previously).

## Case report

**Figure 1.**
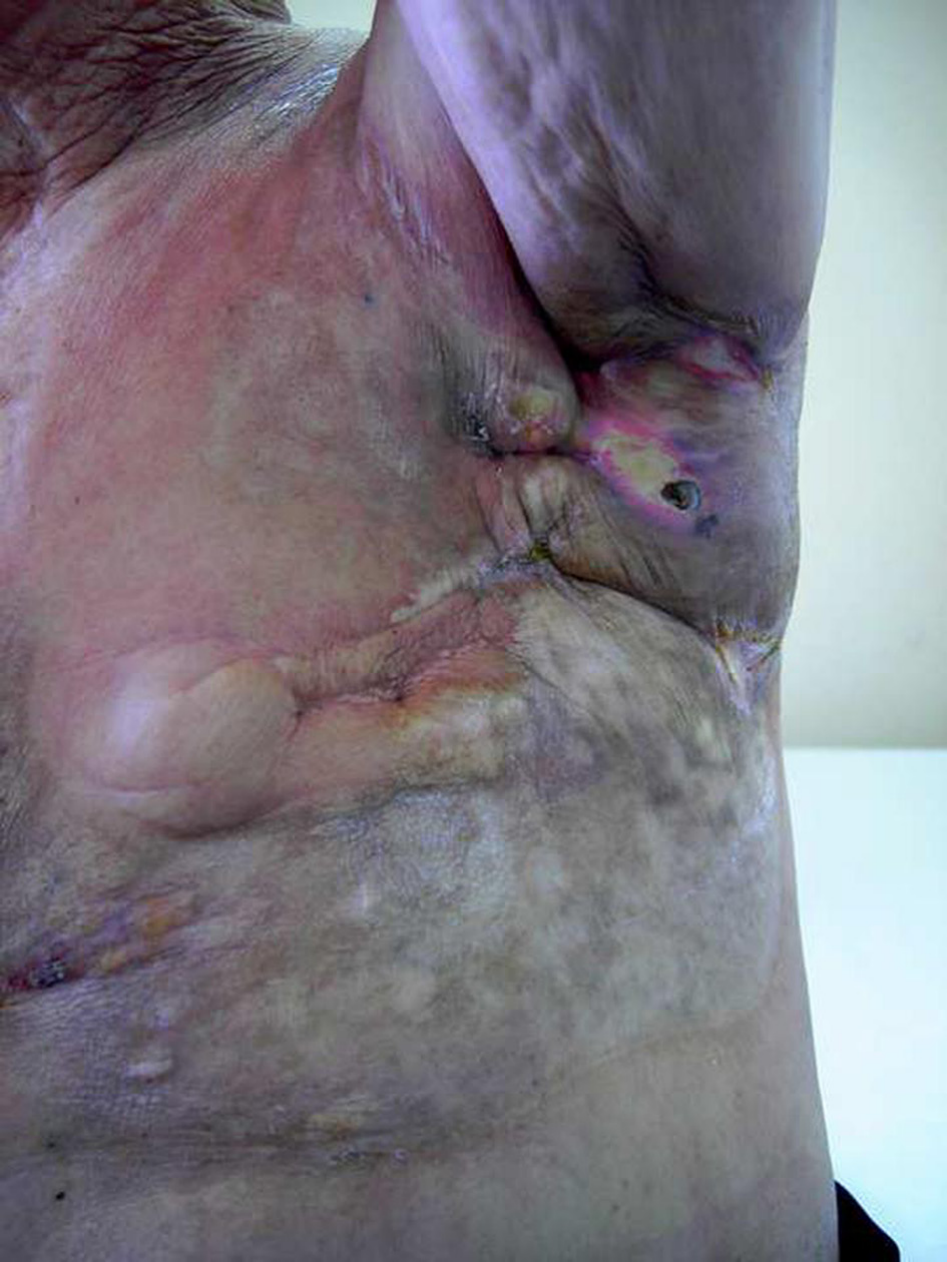
Recall dermatitis: anterolateral view of the thorax.

**Figure 2.**
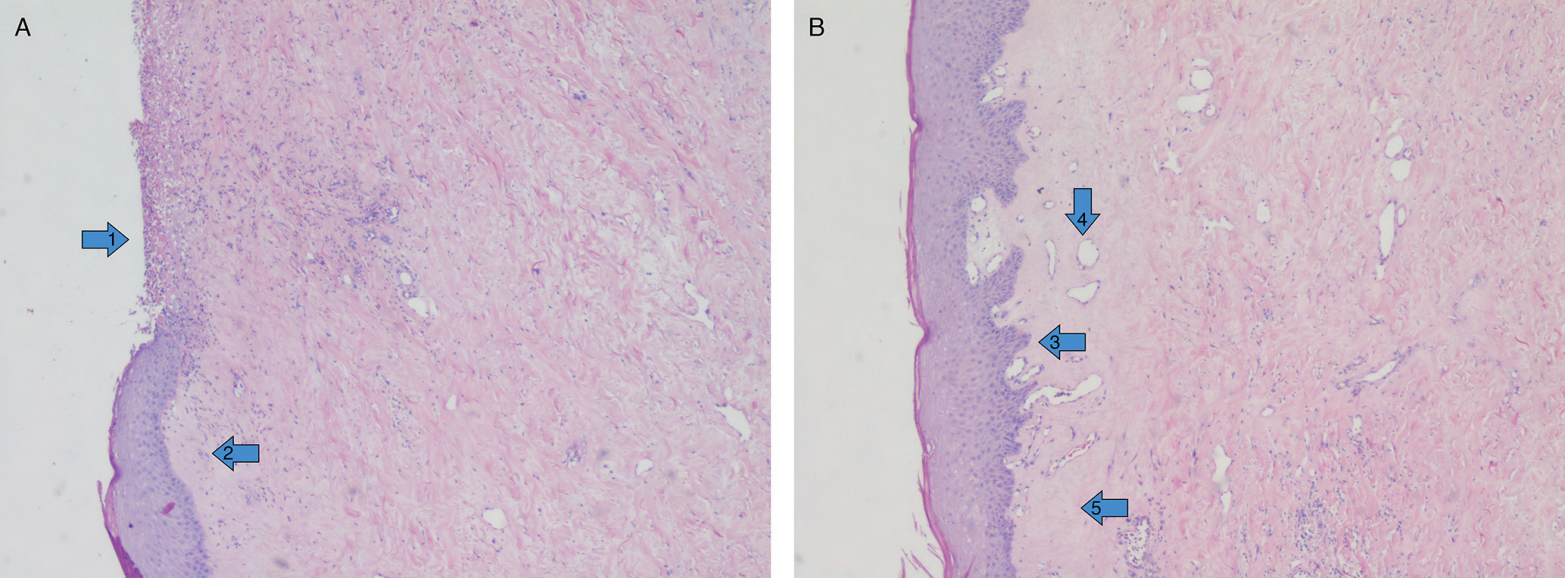
Punch skin biopsy: ulcer clearly present (arrow 1) over granulation tissue. The area with preserved epidermis (arrow 2) shows acanthosis (arrow 3) and parakeratosis with loss of epidermal ridges. Vascular ectasia (arrow 4), hyalinized collagen (arrow 5) and loss of skin adnexa are seen in the dermis. Infiltration by neoplasm was ruled out.

A 78 year old woman allergic to salicylics was diagnosed with a T4dN3M0 ( American Joint Committee on Cancer) infiltrating ductal left breast carcinoma (inflammatory breast cancer) in March 2006. Owing to her general condition and advanced local disease, she was initially treated with primary hormonotherapy consisting of letrozole 2.5 mg/d over a period of six months with a good local response as measured by ultrasound scanning. In October 2006, she was operated on and a modified radical mastectomy was performed. Pathology reported a 6 cm in diameter infiltrating ductal carcinoma pT4dN2a positive for both estrogen and progesterone receptors, and Her2-neu negative. After surgery she started on chemotherapy (Taxol 80 mg/m ^2^ on a weekly schedule for four weeks) and adjuvant radiotherapy (50 Gy over left hemithorax and supraclavicular nodes in February 2007). Immediately after initial radiotherapy, in 2007, she developed skin toxicity Radiation Therapy Oncology Group (RTOG) grade 2, which was successfully managed with topical medication (Radiocrem® Rotthafarm SL [tocopheryl acetate, disodium EDTA, silybum marianum, vitis vinifera] three times a day). She started letrozole 2.5 mg/d again in January 2007. In September 2009 she developed neoplasic left pleural involvement and began hormonotherapy with fulvestrant 500 mg/monthly for five months, followed by exemestane 25 mg/d due to clinical and radiological progression. In May 2010, she developed new pleural progression, which was treated with capecitabine at a dose of 1000 mg/m ^2^/12h (three cycles). Three months later in July 2010, she was noted to have rapidly developed (no more than two weeks after the patient felt the first symptoms of skin stiffness and a local burning sensation) a series of ulcers on the previous mastectomy scar, which had changed in colour (hyper and dispigmentation) and elasticity (stiffness and extreme fragility) over the skin of the previously irradiated area in the left hemithorax ( Figure 1). A punch-biopsy was performed, pathological changes in the skin consistent with radiodermatitis were observed and carcinoma in the involved skin was ruled out ( Figure 2). The diagnosis of a recall radiodermatitis (ulcerating dermatitis, grade 4 RTOG) was thus established in July 2010 and, to mimize the risk of an opportunistic infection, capecitabine was withdrawn and palliative 20 mg tamoxifen started. The skin began to improve after 3–4 weeks following withdrawal of capecitabine and treatment with topical steroids (Menaderm® Menarini; beclomeatsone 0.025 %).

Tamoxifen treatment stabilized the patient’s disease and serological response for four months. Afterwards, pleural progression was diagnosed and vinorelbine started with good response; after eight cycles, the patient suffered a new episode of skin toxicity that was managed with vinorebiline withdrawal and letrozole treatment, which allowed for a nine month stability period. In March 2012, progression was seen (liver metastasis and greater pleural effusion with clinical deterioration) and cyclophosphamide started. All active medication was stopped in May that year and palliative care lasted until the patient passed away a few months afterwards.

## Discussion

Radiation-recall dermatitis is an inflammatory reaction of the skin at the site of previous irradiation. Many chemotherapy drugs have been presumed to cause this phenomenon and a database to collect these rare reaction cases has even been proposed ^1^. It can also affect other anatomical areas such as the digestive system (when abdominal radiotherapy has been used) ^2^. Although a closer time gap is more usual, the time gap between the inflammatory reaction and previous radiation can range from days to several years ^3^.

There are few reported cases of capecitabine-induced radiation recall phenomenon, the first one being authored by Ortmann *et al.* in 2002 ^4^. Their hypothesis relied on the pro-drug entity of capecitabine being capable of being activated in previously irradiated tissue.

More recently, Ghosal and Misra have reported a case ^5^ with thoracic hyperpigmentation instead of inflammation being the main clinical finding. Other well known capecitabine side effects are hyperpigmentation associated with palmar-plantar erythrodisesthesia ^6^ or even Stevens-Johnson syndrome ^7^. Some authors have suggested that skin toxicity might be a predictor of response though it has been better related to anti-epidermal growth factor receptor monoclonal antibodies ^8^.

## Conclusions

To our knowledge, this is the first reported case of capecitabine-induced RTOG grade 4 (ulcerating dermatitis) recall skin toxicity of previously irradiated skin. We suggest it is relevant for differential diagnosis with other entities such as local cancer relapse or even surgical site infection. In case of any doubt, such as in our case, punch biopsy can help. Clinicians should be aware of this phenomenon, even if a long period of time has elapsed since the previous radiation therapy.

## Consent

Written informed consent for publication of the clinical details and clinical images was obtained from the patient.
