# Supplementary material for: Capecitabine-induced radiation recall phenomenon: a case report
Source: F1000Res. 2013 Mar 7;1:64. Originally published 2012 Dec 13. [Version 2] doi: 10.12688/f1000research.1-64.v2 (PMC3869495; doi:10.12688/f1000research.1-64.v2)
Supplement: Peer review history

# Peer review: Referee response for version 2

**Published:** 2013-05-07

**Authors:** Rita De Sanctis

**Affiliations:** 1 Humanitas Cancer Center, Istituto Clinico Humanitas, IRCCS, Rozzano, Italy

I have read this submission. I believe that I have an appropriate level of expertise to confirm that it is of an acceptable scientific standard.

# Peer review: Referee response for version 1

**Published:** 2012-12-19

**Authors:** Rita De Sanctis

**Affiliations:** 1 Humanitas Cancer Center, Istituto Clinico Humanitas, IRCCS, Rozzano, Italy

I give this article an ‘approved with reservations’. The reason for this is that I found that it lacks a few fundamental aspects:

- What was the response after 3 cycles of capecitabine? Please remember (and eventually cite) some reports of skin toxicity as predictor of response.
- It would be interesting to provide a description of the inflammatory characteristics of the punch-biopsy (even with iconografic report, if available).
- What was the presentation of the skin toxicity? Sudden or progressive? Were some topical medications used before the appearance of Figure 1 aspects?
- “Palliative” is all the setting of the present clinical case and not only when the authors decided to withdrawn capecitabine. What is the reason for the choice of tamoxifen instead of another chemotherapy,i.e. vinorelbine?
- Radiocrem (R): please provide the active components and avoid the commercial brand.
- What was the duration of stable disease (“the disease is now stable”).
- Palmar-plantar erythrodisesthesia is not so rare with capecitabine (my group and I have reported a 8% of G3/G4 skin toxicity with capecitabine). Moreover, according to guidelines of side effects management, do the authors believe that a rechallenge with capecitabine after dose reduction could be performed?

I have read this submission. I believe that I have an appropriate level of expertise to confirm that it is of an acceptable scientific standard, however I have significant reservations, as outlined above.

# Response

**Published:** 2013-02-08

**Authors:** Jose Aguilar

**Affiliations:** Hospital Morales Meseguer. Murcia, Spain

Dear Dr De Sanctis,

Thank you for your detailed and relevant comments. We have sent an amended text to the editor that includes the following answers to your questions (and 2 figures from the skin biopsy): 

**1) **After 3 cycles of capecitabine, the disease remained stable. Some authors have suggested that skin toxicity might be a predictor of response, though it has been better related to anti-epidermal growth factor receptor monoclonal antibodies (Petrelli F, *et al*. The predictive role of skin rash with cetuximab and panitumumab in colorectal cancer patients: a systematic review and meta-analysis of published trials. Target Oncol. 2013 Jan 16. [Epub ahead of print] ) http://www.ncbi.nlm.nih.gov/pubmed/23321777

**2)** In the biopsy, ulcers can be easily seen over granulation tissue. The area with preserved epidermis shows acanthosis and parakeratosis with loss of epidermal ridges. Vascular ectasia, hyalinized collagen and loss of skin adnexa is seen in the dermis. Infiltration by neoplasm was ruled out. We have enclosed 2 new figures with these findings to the original text. You can view these in Version 2 of our article, Figure 2.

**3)** Skin toxicity appeared in a rapidly progressive pattern, no more than 2 weeks after the patient felt the first symptoms (skin stiffness and a burning  local sensation) . No topical medication was used before the skin rash appeared.

**4)** The term “palliative” has been used throughout the text to describe chemotherapy in the context of a patient with metastatic disease, with the objective of relieving symptoms and to delay the expected evolution. The possibility of the ulcers being the origin of an opportunistic infection was the reason why we chose the option of tamoxifen treatment instead of further chemotherapy.

**5)**  Radiocrem ® was used to avoid description of the different active components of the cream that include: tocopheryl acetate, disodium EDTA, Silybum marianum, Vitis vinifera, etc.

**6)** Tamoxifen treatment allowed the patient to have 4 months with stable disease and serologic response. Afterwards, pleural progression was diagnosed and vinorebiline treatment was started with a good response; after 8 cycles, the patient suffered a new episode of skin toxicity that was managed with vinorebiline withdrawal and Letrozole treatment, which allowed for a 9 month stability period. On March 2012, progression was seen (liver metastasis and greater pleural effusion  with clinical deterioration) and cyclophosphamide treatment was started. All active medication was stopped in May and palliative care lasted until the patient died a few months afterwards.

**7)** We agree with your statement. “Rare” might not be the best word for this side effect  frequency. Anyway, though palmar-plantar erythrodisesthesia is a well known dose-dependent effect, recall radiodermatitis ****does not seem to have that characteristic. That is why we tried to find another treatment and did not use capecitabine again in this patient.

# Peer review: Referee response for version 1

**Published:** 2012-12-17

**Authors:** Jay Thomas

**Affiliations:** 1 Comprehensive Care Program, HealthCare Partners, Arcadia, USA

I have read this submission. I believe that I have an appropriate level of expertise to confirm that it is of an acceptable scientific standard.
